# News from the Societies

**Published:** 1961-10

**Authors:** 


### *News from Societies*

### BATH CLINICAL SOCIETY

*Secretary:* Mr. T. L. Schofield, "Sunnycroft", Bloomfield Park, Bath

13th Oct.: **Clinical Meeting.**
10th Nov.: Dr. J. J. Ellison, Dr. J. Cosh, Mr. T. Schofield., **Sympsium on Thyroid Disease.**
8th Dec.: Sir W. Russell Brain, D.M., F.R.C.P.
12th Jan.: **Clinical Meeting** at St. Martin's Hospital, Bath.
9th Feb.: Dr. John Naish, **"Management of Acute Ulcerative Colitis and Crohn's Disease."**
9th Mar.: Dr. R. McNulty, Dr. E. Hickson.
13th Apr.: **Annual General Meeting.** Dr. Hugh Wallis or Dr. John Bolton.
All meetings will be held at the Royal United Hospital unless otherwise stated.

### BRISTOL MEDICO-CHIRURGICAL SOCIETY

*Secretary:* Dr. A. T. M. Roberts, 12 Southfield Road, Westbury-on-Trym, Bristol

11th Oct.: **Presidential Address and Annual General Meeting.** (President-elect Mr. J. Angell James).
8th Nov.: **Clinical Meeting** at B.R.I.
13th Dec.: Terence E. Cawthorne, F.R.C.S. (President's Guest) will address the Society.
9th Jan.: Professor J. A. Coutts, Professor of Law in the University of Bristol, will address the Society.
14th Feb.: Dr. N. J. Brown and Dr. Beryl Corner will read papers.
14th Mar.: Mr. H. K. Bourns will read a paper.
11th Apr.: Dr. R. P. Warin will read a paper.
9th May: Mr. Keith Lucas will read a paper.
All meetings will be held in the Physics lecture theatre at the Royal Fort unless otherwise stated.

### UNIVERSITY OF BRISTOL

### POSTGRADUATE COURSES

The following course is being arranged:
Sunday morning Lecture-demonstrations, from 8th October to 3rd December, 1961.
Details may be obtained from the Department of Postgraduate Medical Studies.

### B.M.A. (Bristol Division)

*Hon. Secretaries:* Dr. A. S. Anderson, 10 Leigh Road, Bristol 8, and Dr. H. Temple-Phillips, Berkeley House, Charlton Road, Westbury-on-Trym, Bristol

18th Oct., at 8.30 p.m.: **Annual General Meeting** in Queen's Building, University of Bristol.
22nd Oct., at 2.45 p.m.: **St. Luke's Tide Service** at Bristol Cathedral.
7th Nov., at 8 p.m.: **Annual Dance** at the Berkeley Cafe.
Other meetings to be arranged.

### B.M.A. (Cornwall Division)

*Hon. Secretary:* Dr. Eric Townsend, Fieldways, Tregenna Lane, Camborne

4th Nov.: **Meeting of Branch Council,** S.W. Branch B.M.A. and Clinical Meeting. St. Lawrence's Hospital, Bodmin.
23rd Nov.: **Annual Dinner and Dance,** Hotel Bristol, Newquay.
13th Apr., at 8.30 p.m.: **Annual B.M.A. Clinical Lecture** by Professor A. Moncrieff, Royal Cornwall Infirmary, Truro.
2nd June: **Annual Meeting** S.W. Branch B.M.A., Newquay.

### B.M.A. (Gloucestershire Branch)

*Hon. Secretary:* Dr. H. G. Scott-Kerr, 29 Leckampton Road, Cheltenham

12th oct., at Arle Court, Cheltenham. Presidential Address, **"The General Practitioner in Industrial Medicine".**
9th Nov., in Gloucester: Representatives' Report. Financial Statement. **Paper—"The Medical Care of the Elderly",** by T. W. Lloyd.

14th Dec.: at The Pump Room, Cheltenham. **Address, "Crime and the Doctor",** (illustrated with slides), by Dr. Keith Simpson, Reader in Forensic Medicine in the University of London. A joint meeting with the Gloucestershire members of the Gloucestershire and Wiltshire incorporated Law Society and other members of the legal profession.

11th Jan., in Gloucester: **Paper, "Surgical Complications of Pregnancy"**, by Mr. David Saltau.

8th Feb., in Cheltenham, at 8.15 p.m.: **"The Emergency Bag".** A discussion with a panel of four consultants.

8th Mar., in Gloucester: **Clinical Meeting.**

5th Apr., at the Pump Room, Cheltenham: B.M.A. **Lecture** by Glyn Edmund Daniel, Esq., M.A., Ph.D., Fellow of St. John's College Cambridge, on **"The Antiquity of Man".** Guest night, doctors' ladies and guests invited.

10th May, in Cirencester: **Annual Meeting.** Paper, "Skin deep", by Dr. R. Bowers.

14th June, in Cheltenham: The President's guest.

Unless otherwise stated meetings will be held at 6.15 p.m.

The place of meetings will be stated in the circular notices.

### B.M.A. (South-Western Branch)

*Hon. Secretary:* Dr. F. E. Graham-Bonnalie, 37 The Strand, Topsham, nr. Exeter

26th Oct., **Joint Meeting and Dinner,** Exeter and District Medico-Chirurgical Society and the B.M.A. Exeter Division. The Library, Royal Devon and Exeter Hospital at 4 p.m. **Dinner** at the Imperial Hotel, Exeter, at 7.30 p.m.

4th Nov., at 2 p.m.: **Meeting** of the S. W. Branch of the B.M.A. at Bodmin.

9th Nov., at 8.15 p.m.: **Joint Social and Scientific Meeting,** Exeter Division, Library, Royal Devon and Exeter Hospital, Exeter.

9th or 16th Dec.: B.M.A. Exeter Division, **Annual Dance** at the Imperial Hotel, Exeter, preceded by a **Dinner** at 7.30 p.n.

### CORNWALL CLINICAL SOCIETY

*Hon. Secretaries:* Dr. H. S. Bennett, Royal Cornwall Infirmary, Truro, and Mr. P. N. Simons, Camborne-Redruth Hospital, Redruth

13th Oct.: **Clinical Evening.** Redruth Hospital.

9th Nov.: **"Rheumatoid Arthritis".** Dr. Leslie Hill, Royal Cornwall Infirmary, Truro.

7th Dec.: **"Flatulence".** Mr. Norman Tanner, Royal Cornwall Infirmary, Truro.

11th Jan.: **Clinical Evening.** Royal Cornwall Infirmary, Truro.

9th Feb.: **Clinical Evening.** Tehidy Hospital.

8th Mar.: **"The Medical Aspects of Oscar Wilde".** Dr. Macdonald Critchely, Royal Cornwall Infirmary, Truro.

4th May: **Clinical Evening.** Redruth Hospital.

7th June: **Presidential Address and Annual General Meeting.** Royal Cornwall Infirmary, Truro.

### DEVON AND EXETER MEDICO-CHIRURGICAL SOCIETY

*Hon. Secretary:* Dr. F. S. W. Brimblecombe, 6 Barnfield Crescent, Exeter

12 Oct., at 3 p.m.: **Clinical Meeting.**

26th Oct., at 3.30 p.m.: **"Skin Manifestations of Systematic Disease",** by Dr. H. J. Wallace. **Combined meeting** with the Exeter Division of the B.M.A. Exeter and South Western Medical Dinner.

15th Nov., at 8.15 p.m.: Clinico-pathological demonstration, arranged by Dr. George Stewart Smith.

7th Dec., at 8.15 p.m.: **"Cervical Disc Lession",** by Dr. James Cyriax.

11th Jan., at 8.15 p.m.: **"The Problems of Arthritis in Practice". Addressed by the President,** Dr. N. Sawers. Discussion to be opened by Dr. A. F. H. Coke of the Charterhouse Rheumatism Clinic.

1st Feb., at 3 p.m.: **Clinical Meeting.**

22 Feb., at 8.15 p.m.: **"The Management of Femoral Vein Thrombosis and Pulmonary Embolus".** Openers—Dr. G. Stewart Smith, Dr. Anthony Daly, H. Dendy Moore, Esq., J. Lloyd Griffiths, Esq.

29th Mar., at 8.15 p.m.: **Annual General Meeting.** Clinico-pathological discussion arranged by Dr. G. Stewart Smith.

10th May, at 8.15 p.m.: **Clinical Meeting** at the Princess Elizabeth Orthopaedic Hospital.

### ROYAL DEVON AND EXETER HOSPITAL POST GRADUATE COURSE

*Hon. Secretary:* Mr. H. Dendy Moore, 4 Magdalen Road, Exeter

10th Oct.: **"Pulmonary Tuberculosis—Medical Treatment".** Dr. B. R. Hills. **"Pulmonary Tuberculosis—Surgical Treatment".** Mr. L. Lloyd Griffiths.
17th Oct.: **"Geniti-Urinary Tyberculosis".** Mr. D. Innes Williams.
24th Oct.: **"Bone and Joint Tuberculosis".** Mr. Norman Capener.
31st Oct.: **"Indications for Brain Surgery in Childhood".** Dr. F. S. W. Brimblecombe.
7th Nov.: **"Indications for Neurosurgery in Adults".** Dr. N. S. Alcock.
14th Nov.: **"Some Aspects of Neurosurgery".** Mr. Kenneth Till.
21st Nov.: **"Gall Stones".** Mr. P. Monks.
28th Nov.: **"Pancreatitis—Acute and Chronic".** Mr. David I. Stirk.
5th Dec.: **"Alimentary insufficiency".** Dr. A. J. Daly.
Meetings will be held at 8.30 p.m. in the Library of the Royal Devon and Exeter Hospital.

### SOCIETY OF MEDICAL OFFICERS OF HEALTH (West of England Branch)

*Secretary:* Dr. J. F. Skone, Department of Public Health, Tower Hill, Bristol 2

The Society is divided into two sections, Northern and Southern. Each section holds four meetings a year, one of which being a joint meeting of both sections.
7th Oct.: R. H. G. H. Denham, Esq., M.D., D.P.H., M.O.H. Bathavon, Frome and Keynsham District Councils. **Presidential Address** (Taunton).
6th Jan.: At Bristol (details to follow).
April: **Joint Meeting** of Northern and Southern sections (details to follow).
June: At Bath (details to follow).

### SOUTH SOMERSET MEDICAL SOCIETY

*Hon. Secretary:* Dr. S. T. Pickles, 68 Hendford, Yeovil, Somerset

19th Oct.: **Annual General Meeting.** Presidential Address by J. C. McMaster, M.D., F.R.C.S., **"A Story of Occupation".**
16th Nov.: **"Epilepsy and Mechanisms of Consciousness",** by J. W. Warboys, M.D., D.P.M.
14th Dec.: **Dinner and Dance.**
18th Jan.: D. G. Vulliamy, M.D., M.R.C.P., D.C.H., on some peadiatric subject.
15th Feb.: **"Manipulative Methods in Orthopaedics",** by J. F. Bourdillon, F.R.C.S.
15th Mar.: F. J. Wood, M.D., M.R.C.P. probably on congenital heart disease.

### TORQUAY AND DISTRICT MEDICAL SOCIETY

*Hon. Secretaries:* Dr. D. K. McTaggart, Health Department, St. Marychurch Town Hall, Torquay, and Dr. J. B. Taylor, Corner Place, Dartmouth Road, Paignton

19th Oct.: **"Adreno-Cortical Syndromes",** by Dr. Alan Spence.
2nd Nov.: **"Patterns of Injury in Forsenic Medicine",** by Dr. Donald Teare.
16th Nov.: **"Recent Advances in Tissue Histology",** by Professor Gilbert Causey.
7th Dec.: **"Brains Trust"** Panel: Mr. P. J. W. Monks, Dr. R. L. Midgely, Dr. L. Haas.

### WESSEX RAHERE CLUB

The autumn dinner of the above club will take place on 28th October, 1961 at the Lansdown Grove Hotel, Bath, under the chairmanship of Dr. Pascoe Hill, of Bristol. The Guest of Honour, it is hoped, will be Professor Michael Boyd. Further details will be circulated or can be obtained by any Barts. graduates who are not already members from the Hon. Secretary, Mr. A. Daunt Bateman, F.R.C.S., 11 The Circus, Bath.

### WEST OF ENGLAND CHILD HEALTH GROUP

*Hon. Secretary:* Dr. A. L. Smallwood, Department of Public Health, Tower Hill, Bristol 2

12th Oct., 8.30 p.m.: **"Teenages Forum",** Dr. A. J. Dalzell-Ward, Director, Central Council for Health Education, and Team.
22nd Nov., 5.30 p.m.: **"Rural Paediatrics",** Dr. Brian Webb.
13th Dec., 5.30 p.m.: **"Health of Immigrants",** Dr. J. F. Skone.
24th Jan., 8.30 p.m.: **"Turkish Delight—Health in the Eastern Mediterranean",** Dr. Beryl Corner, Miss N. Deane and Miss A. I. Rowbottom.
28th Feb., 5.30 p.m.: **"An Epidemic of Tuberculosis",** Dr. R. A. Craig, Dr. G. F. Bramley.
28th Mar., 5.30 p.m.: **"Experiences of a Medical Officer, Student Games, Bulgaria, 1961",** Dr. Marrian.
12th May: **"Clinical Meeting",** at the Royal United Hospital, Bath.
All meetings will take place in the Department of child Health Lecture Theatre, Children's Hospital, Bristol, unless otherwise stated.

